# 682. Impact of Antibiotic Time-Out Rounds on Academic Internal Medicine Services

**DOI:** 10.1093/ofid/ofaf695.221

**Published:** 2026-01-11

**Authors:** Bradley Lauver, Caroline E Dillon, Jamie Hood, Alejandra Perez-Chapman, Stanley I Martin, Lauren DiMarino

**Affiliations:** Geisinger Medical Center, Danville, Pennsylvania; Geisinger Medical Center, Danville, Pennsylvania; Geisinger Medical Center - - Danville, PA, Danville, Pennsylvania; Geisinger Medical Center, Danville, Pennsylvania; Geisinger, DANVILLE, Pennsylvania; Geisinger, DANVILLE, Pennsylvania

## Abstract

**Background:**

Research indicates various antimicrobial stewardship (ASP) strategies have been effective within medical residency programs^1,2^. Involving internal medicine (IM) residents in ASP is essential for optimizing antibiotics, reducing resistance, and improving patient outcomes. We introduced Antibiotic Time-Out (ATO) in our academic IM service to promote clinical outcomes and positively influence prescribing habits.

Table 1

**Figure:**
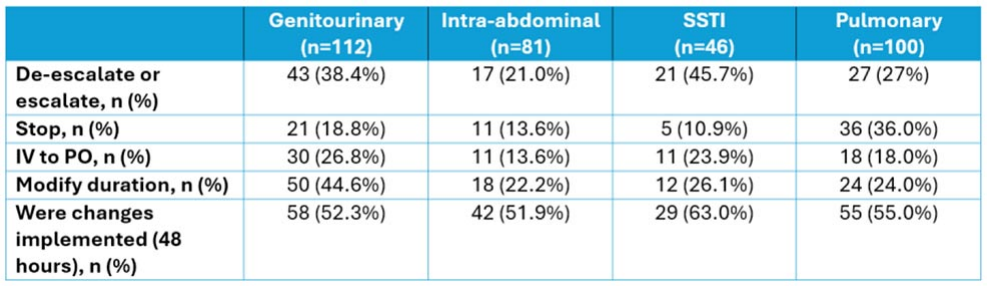

**Figure 1:**
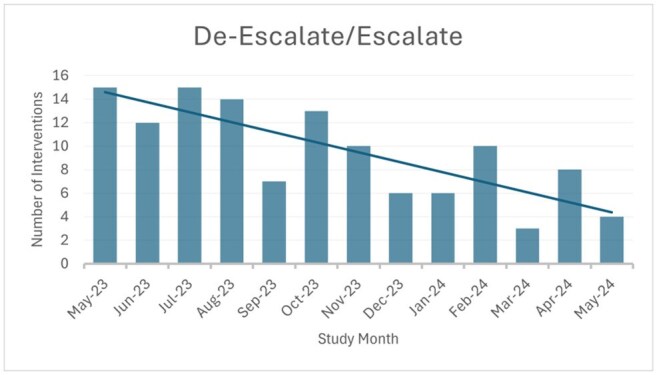
De-Escalate/Escalate Intervention Over Time

**Methods:**

The ATO initiative (May 2023-May 2024) aimed to discuss all patients on antimicrobials not already followed by the Infectious Disease (ID) consult service. Patients were presented to an ID physician and pharmacist, who made recommendations such as clarifying diagnoses, performing additional workup, changing antibiotics, adjusting the route or duration, or obtaining formal ID consultation if applicable. Patients were reviewed retrospectively to determine if the recommended change was made within 48 hours.

**Figure 2:**
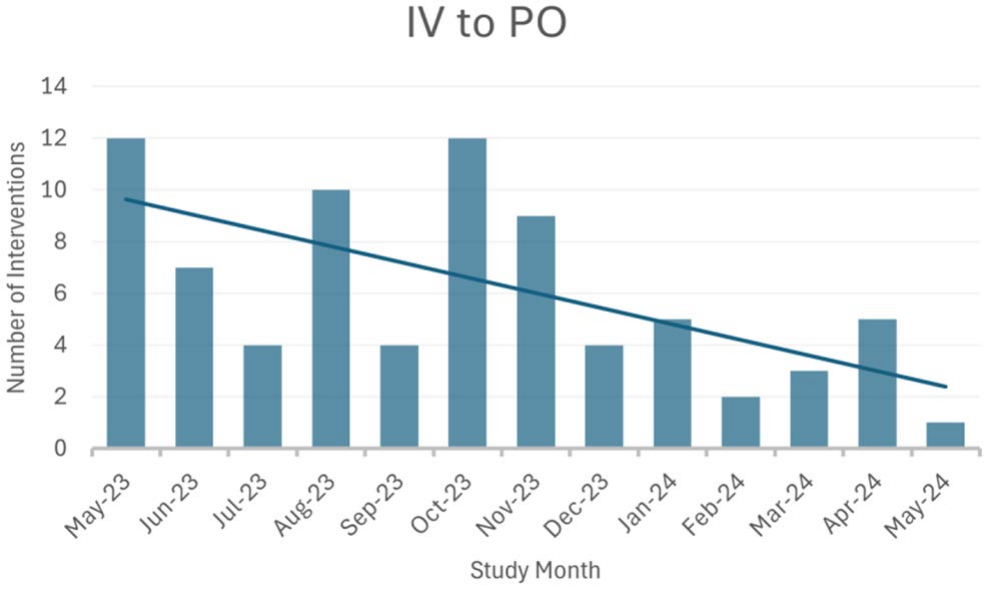
IV to PO Intervention Over Time

**Figure 3:**
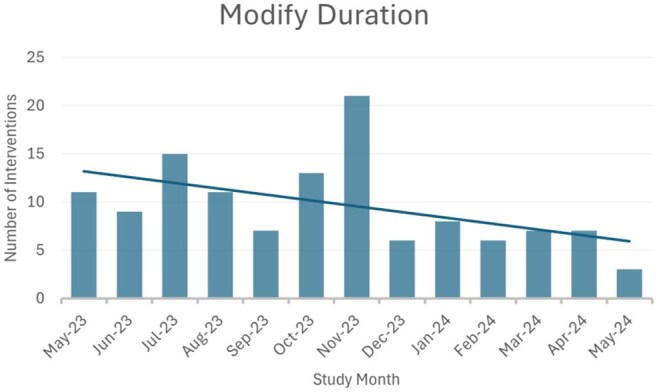
Modify Duration Intervention Over Time

**Results:**

A review of 393 patients resulted in 446 recommendations, the most common of which included de-escalation/escalation (31.3%, n=123), stop therapy (21.4%, n=84), and modify duration (31.6%, n=124). Overall, 83.2% of recommendations were implemented within 48 hours. Of the 123 patients where spectrum modifications were recommended, 43.9% (n=54) resulted in de-escalation from high-risk *C. difficile* (CDI) antibiotics, 52.8% (n=65) led to the elimination of anti-*Pseudomonal* therapy, and 66.6% (n=82) resulted in increased use of a narrow-spectrum β -lactam. Indication specific results can be found in Table 1. In addition, we observed improved trends in clinical practice over time such as reduced need for de-escalation, stepdown to oral therapy, and duration modification. This suggests ATO enhances resident education leading to improved antimicrobial prescribing practices. See Figures 1-3.

**Conclusion:**

The ATO produced meaningful clinical outcomes of decreased use of anti-*Pseudomonal* and high CDI risk agents, and increased utilization of narrow spectrum β-lactams. The ATO also enhanced educational experiences and improved residents' understanding of ASP principles. This has led to fewer recommendations over time and improvements in empiric antibiotic choices, use of oral therapy, and treatment duration.

**Disclosures:**

All Authors: No reported disclosures

